# Circ_0081001 down-regulates miR-494-3p to enhance BACH1 expression and promotes osteosarcoma progression

**DOI:** 10.18632/aging.203207

**Published:** 2021-06-30

**Authors:** Shizhang Liu, Keke Duan, Xiaoxia Zhang, Xiane Cao, Xixia Wang, Fanbin Meng, Huitong Liu, Bingqiang Xu, Xi Wang

**Affiliations:** 1 Department of Orthopedics, Shaanxi Provincial People’s Hospital, Xian 710068, Shanxi, China; 2 Department of Orthopedics, Linyi People's Hospital, Linyi 276100, Shandong, China; 3 Department of Central Sterile Supply, Linyi People's Hospital, Linyi 276100, Shandong, China

**Keywords:** osteosarcoma, circ_0081001, miR-494, BACH1

## Abstract

The study was aimed at deciphering the function and mechanism of circ_0081001 in osteosarcoma (OS). In this study, quantitative real-time polymerase chain reaction (qRT-PCR) was utilized for quantifying circ_0081001, miR-494-3p, and BTB domain and CNC homolog 1 (BACH1) mRNA expressions in OS tissues and cells. Cell counting kit-8 (CCK-8) assay, together with 5-Ethynyl-2'-deoxyuridine (EdU) assay, was performed for evaluating cell proliferation; the alterations in apoptosis were analyzed utilizing flow cytometry; Transwell assay was conducted for examining cell migration and invasion; moreover, Western blot was utilized for the quantification of BACH1 protein expression; bioinformatics, dual-luciferase reporter gene, and RNA-binding protein immunoprecipitation assays were executed for validating the binding relationships between circ_0081001 and miR-494-3p, and between miR-494-3p and BACH1. As shown, circ_0081001, whose expression was elevated in OS tissues, had a negative association with miR-494-3p expression and a positive correlation with BACH1 expression. After circ_0081001 was overexpressed, the proliferation, migration, and invasion of OS cells were boosted but the apoptosis was reduced, whereas miR-494-3p exhibited opposite effects. The binding sites between circ_0081001 and miR-494-3p, and between miR-494-3p and the 3’UTR of BACH1 were experimentally verified. In conclusion, circ_0081001/miR-494-3p/BACH1 axis promoted the malignant biological behaviors of OS cells.

## INTRODUCTION

Osteosarcoma (OS) is identified as a common malignancy amongst children and teenagers [1]. Despite the great progress in the therapeutics of OS, the prognosis remains unsatisfactory due to distant metastasis and recurrence [2]. Towards this end, it is vital to better deepen the mechanism of OS growth, as it will aid in the development of novel therapeutic strategies [3].

Circular RNAs (circRNAs), a subgroup of transcripts with circular structures, are stable in tissues and cells. Reportedly, they are crucial regulators of gene expression [4–10]. Circ_0102049, for example, promotes OS progression via modulating miR-1304-5p/MDM2 axis [8]. Circ_0001146 represses miR-26a-5p expression and up-regulates MNAT1 expression in OS cells, hence impeding OS growth [9]. Reportedly, circ_0081001 expression is elevated in OS tissues, cell lines, as well as patients' serum; COX regression analysis indicates that circ_0081001 is an independent risk factor that predicts the poor prognosis of the patients with OS [10]. Nonetheless, its biological functions and underlying mechanism in OS warrant further investigation.

At the post-transcriptional level, microRNAs (miRNAs) suppress gene expression [11]. Besides, miRNAs can regulate proliferation, apoptosis, metastasis, and metabolism of cancer cells in multiple cancers [12–15]. MiR-494-3p features prominently in the progression of multiple cancers. MiR-494-3p, for example, whose expression is down-regulated in ovarian cancer, constrains cell proliferation and facilitates apoptosis [14]. In OS, miR-494-3p impedes the above malignant biological behaviors of OS cells by repressing insulin receptor substrate-1 [15].

This work substantiated that circ_0081001, functioning as competitive endogenous RNA (ceRNA), promoted the malignancy of OS cells via repressing miR-494-3p expression and up-regulating BTB domain and CNC homolog 1 (BACH1) expression.

## RESULTS

### Bioinformatics analysis suggested that circ_0081001/miR-494-3p/BACH1 could probably participate in OS progression

**Figure 1:**
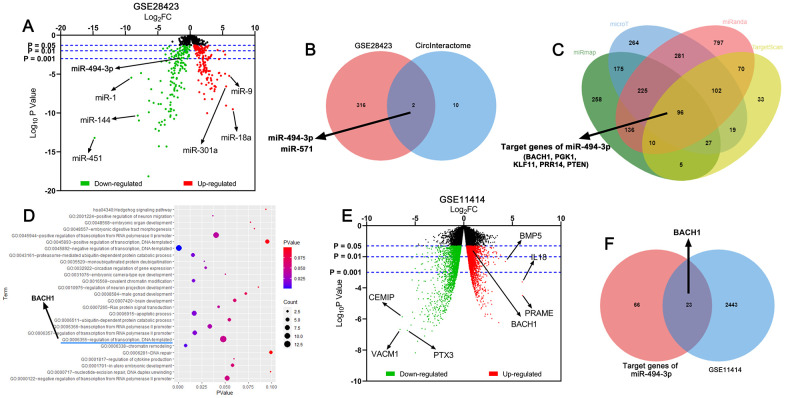
**Circ_0081001, miR-494-3p, and BACH1 were potential regulators in OS.** (**A**) Volcano map was used to indicate the difference of the expression levels of miRNAs between normal bone tissues and OS tissues in GSE28423, and the upregulated and downregulated miRNAs expressions were marked with red and green, respectively. (**B**) Venn diagram showed the intersection of predicted miRNA targets of circ_0081001 and lowly expressed miRNAs in OS tissues in GSE28423. (**C**) MiRmap, microT, miRanda, and TargetScan were employed for predicting the downstream target genes of miR-494-3p. (**D**) The potential biological function of miR-494-3p was predicted utilizing DAVID GO enrichment analysis. (**E**) Volcano map was used to indicate the difference of the gene expression profile between human normal osteoblasts and OS cell line in GSE11414, and the upregulated and downregulated gene expressions were marked with red and green, respectively. (**F**) Venn diagram showed the intersection of predicted target genes of miR-494-3p and highly expressed genes in OS tissues in GSE11414.

By analyzing the miRNA expression profile in GSE28423, the down-regulation of miR-494-3p expression was uncovered in OS tissues as against in normal bone tissue, and meanwhile, miR-494-3p was predicted to be a downstream target of circ_0081001 by CircInteractome database (Figure 1A, 1B). Besides, the results unmasked that BACH1 was one of the downstream target genes of miR-494-3p (Figure 1C). Gene ontology (GO) enrichment analysis was utilized for predicting the potential biological function of miR-494-3p, and BACH1 also partook in regulating DNA-templated, which might influence the growth of the tumor cells (Figure 1D). Interestingly, through analyzing GSE11414, it was revealed that BACH1 expression, among the potential targets of miR-494-3p, was elevated in OS cell line in comparison to in normal human osteoblasts (Figure 1E, 1F). Collectively, a scientific hypothesis that in OS, circ_0081001/miR-494-3p/BACH1 axis could probably regulate OS progression was proposed.

### The expressions of circ_0081001, miR-494-3p, and BACH1 in OS were dysregulated

**Figure 2:**
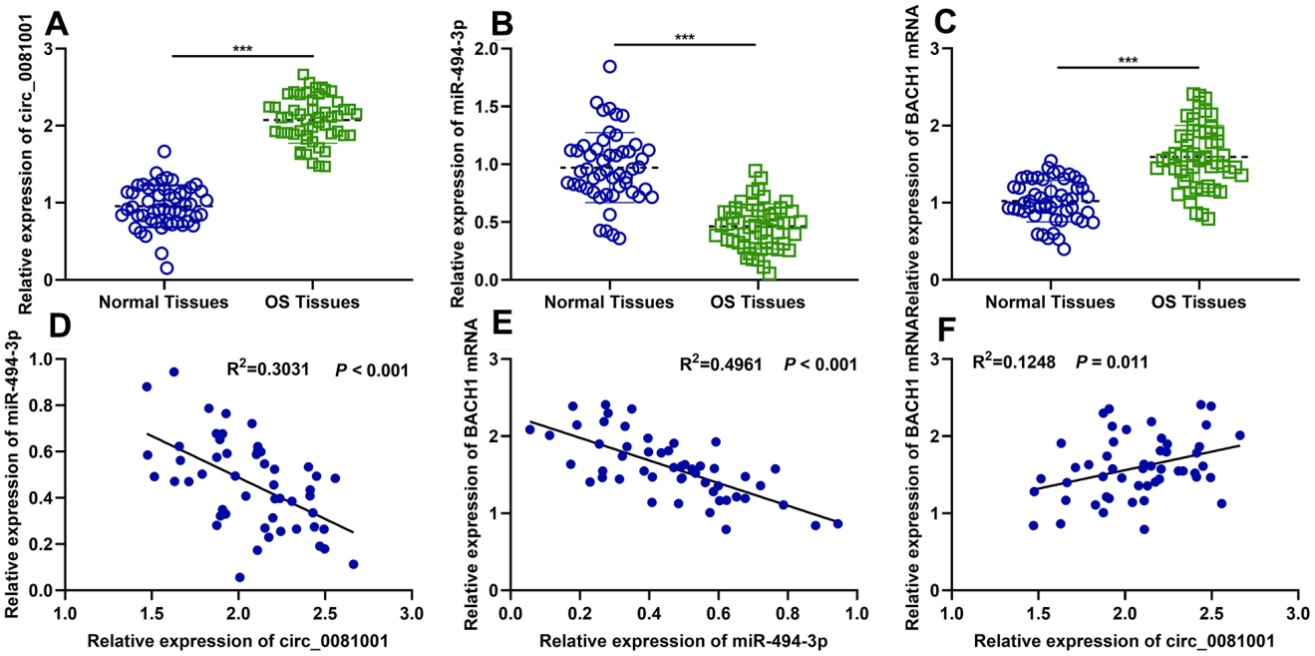
**The expressions of circ_0081001, miR-494-3p, and BACH1 mRNA in OS samples and their correlations.** (**A**–**C**) qRT-PCR was employed for assessing expressions of circ_0081001, miR-494-3p, and BACH1 mRNA in OS tissues and adjacent tissues. (**D**–**F**) The correlations amongst circ_0081001, miR-494-3p, and BACH1 mRNA in OS samples were analyzed by Pearson correlation analysis. ***P<0.001.

Next, circ_0081001, miR-494-3p, and BACH1 mRNA expressions in OS samples were quantified by qRT-PCR, and it was indicated that circ_0081001 and BACH1 mRNA expressions were elevated but miR-494-3p expression was reduced in OS tissues (Figure 2A–2C). Additionally, negative correlations between miR-494-3p and circ_0081001 expressions, as well as between miR-494-3p and BACH1 mRNA expressions were observed, while circ_0081001 expression was positively associated with BACH1 mRNA expression (Figure 2D–2F), further implying the regulatory relationships among them. Additionally, according to the expression of circ_0081001, OS patients were stratified into high (n=26) and low (n=25) circ_0081001 expression group, and it was demonstrated that circ_0081001 was linked to lymph node metastasis and higher TNM stage of the patients (Table 1).

**Table 1:** The correlation between circ_0081001 expression and the pathological characteristics of the patients.

**Variable**	**N**	**Circ_0081001 expression**		***P* value**
		**Low=25**	**High=26**	
Age(years)				
≤20	38	18	20	0.687
>20	13	7	6	
Gender				
Male	24	12	12	0.895
Female	27	13	14	
Tumor size				
≥3	26	12	14	0.782
<3	25	13	12	
Location				
Tibia/femur	42	23	19	0.076
Elsewhere	9	2	7	
Lymph node metastasis				
Positive	32	12	20	0.033*
Negative	19	13	6	
Differentiated degree				
High/middle	38	17	21	0.296
Low/undifferentiation	13	8	5	
TNM stage				
I-II	19	13	6	0.033*
III-IV	32	12	20	

*P<0.05.

### Circ_0081001 regulated the proliferation, migration, invasion, and apoptosis of OS cells

**Figure 3:**
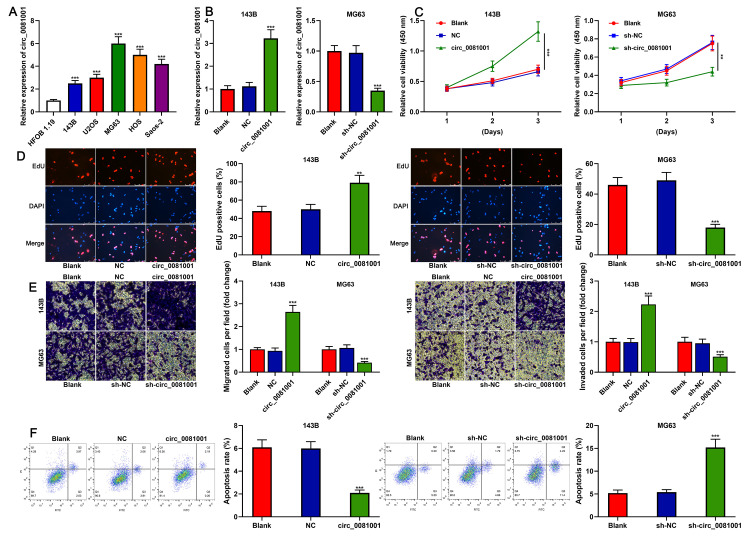
**The effects of circ_0081001 on proliferation, migration, invasion, and apoptosis of OS cells.** (**A**) qRT-PCR was used for detecting circ_0081001 expression in OS cell lines and HFOB 1.19 cells. (**B**) qRT-PCR was used for assaying the transfection efficiency of circ_0081001 overexpression plasmid and sh-circ_0081001 in OS cells. (**C**) CCK-8 method was employed for detecting the viability of 143B and MG63 cells. (**D**) EdU method was employed for examining the proliferation of 143B and MG63 cells. (**E**) Transwell assay was employed for examining the migration and invasion of 143B and MG63 cells. (**F**) The apoptosis of 143B and MG63 cells was evaluated by flow cytometry. ** and *** represent *P*<0.01 and *P*<0.001, respectively.

qRT-PCR elucidated that circ_0081001 expression, compared to that in normal osteoblast HFOB 1.19, was remarkably up-regulated in human OS cell lines (Figure 3A). 143B cell line with the lowest expression of circ_0081001 and MG63 cell line with the highest expression of circ_0081001 were selected for the construction of the cell model of circ_0081001 overexpression and circ_0081001 knockdown, respectively (Figure 3B). CCK-8 and EdU experiments indicated that circ_0081001 overexpression facilitated the proliferation of 143B cells whereas circ_0081001 knockdown suppressed the proliferation of MG63 cells (Figure 3C, 3D). Furthermore, through Transwell experiment, it was demonstrated that circ_0081001 overexpression facilitated the migration and invasion of 143B cells whereas its knockdown worked oppositely on MG63 cells (Figure 3E). Flow cytometry experiment substantiated that circ_0081001 overexpression triggered a significant reduction of apoptosis rate of 143B cells, while circ_0081001 knockdown induced the apoptosis of MG63 cells (Figure 3F).

### miR-494-3p was a target of circ_0081001

**Figure 4:**
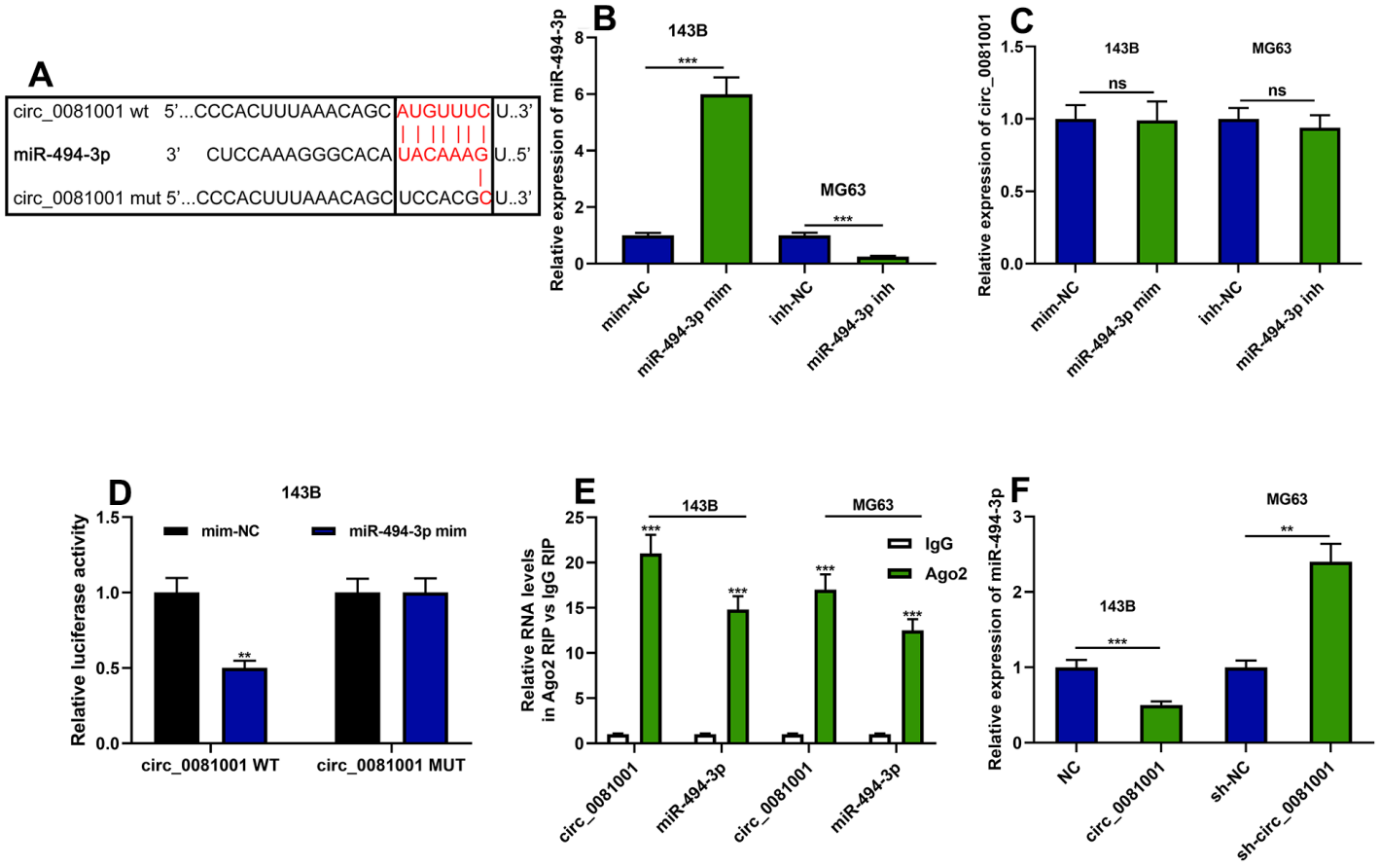
**The targeted relationship between circ_0081001 and miR-494-3p.** (**A**) The potential binding site between miR-494-3p and circ_0081001 was predicted by bioinformatics analysis, and the binding site between miR-494-3p and circ_0081001 was shown. (**B**) qRT-PCR was implemented for examining the transfection efficiency of miR-494-3p mimics and miR-494-3p inhibitors. (**C**) The effects of miR-494-3p on circ_0081001 expression in OS cells were examined with qRT-PCR. (**D**) Dual-luciferase reporter gene experiment was employed for examining the targeted relationship between circ_0081001 and miR-494-3p. (**E**) RIP assay was performed for validating the direct interaction between circ_0081001 and miR-494-3p. (**F**) The impacts of circ_0081001 on miR-494-3p expression in OS cells were detected by qRT-PCR. ** and *** represent *P*<0.01 and *P*<0.001, respectively. ns represents *P*>0.05.

Circ_0081001 was predicted to contain a conserved binding site for miR-494-3p by CircInteractome database (Figure 4A). miR-494-3p mimics and miR-494-3p inhibitor were transfected into 143B and MG63 cells, respectively, to validate the binding sites between circ_0081001 and miR-494-3p (Figure 4B). qRT-PCR suggested that the selective regulation of miR-494-3p exerted no effects on circ_0081001 expression (Figure 4C). Besides, miR-494-3p mimics repressed luciferase activity of luciferase reporter vector containing circ_0081001-WT but no effect on the luciferase activity of circ_0081001-MUT vector was observed (Figure 4D). Further, through RIP assay, it was validated that miR-494-3p directly interacted with circ_0081001 in OS cells (Figure 4E). Besides, circ_0081001 overexpression markedly suppressed miR-494-3p expression in 143B cells while knockdown of circ_0081001 in MG63 cells exhibited an opposite effect (Figure 4F).

### The effects of circ_0081001 on regulating the proliferation, migration, invasion, and apoptosis of OS cells were dependent on miR-494-3p

**Figure 5:**
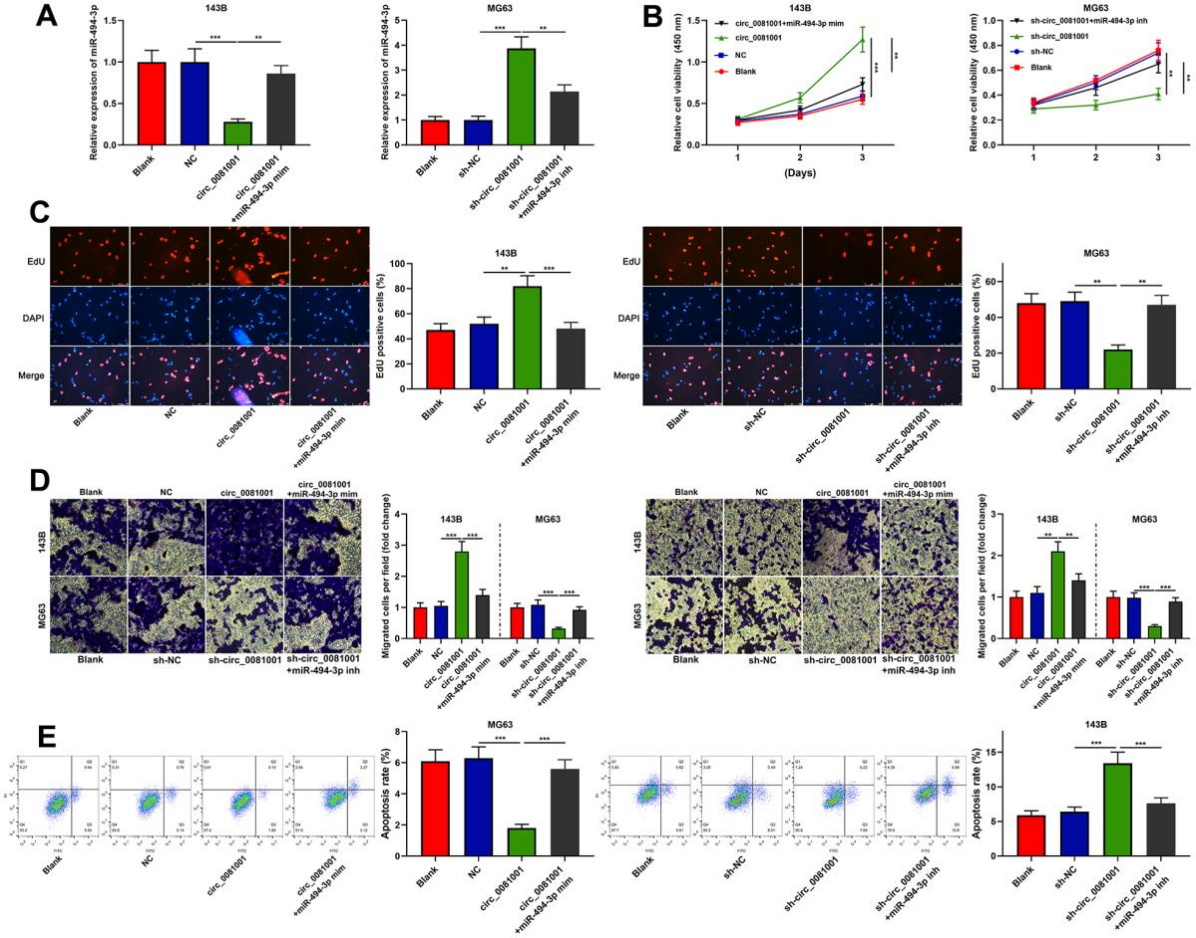
**Circ_0081001/miR-494-3p axis regulated the proliferation, migration, invasion, and apoptosis of OS cells.** Circ_0081001 overexpression plasmid and miR-494-3p mimics were co-transfected into 143B cells, and sh-circ_0081001 and miR-494-3p inhibitors were co-transfected into MG63 cells. (**A**) qRT-PCR was implemented for the detection of transfection efficiency. (**B**) The cell viability of OS cells was examined by CCK-8 assay after the transfection. (**C**) EdU assay was used for detecting cell proliferation of OS cells after the transfection. (**D**) Transwell assay was employed for detecting the alterations of migration and invasion of 143B and MG63 cells after the transfection. (**E**) The apoptosis of OS cells was determined by flow cytometry after the transfection. ** and *** represent *P*<0.01 and *P*<0.001, respectively.

To pinpoint the functions of circ_0081001/miR-494-3p axis on OS cells, miR-494-3p mimics and circ_0081001 overexpression plasmid were co-transfected into 143B cells, and miR-494-3p inhibitors and sh-circ_0081001 were co-transfected into MG63 cells (Figure 5A). The experimental data revealed that miR-494-3p mimics could partially counteract the promotion induced by circ_0081001 overexpression on the malignant biological behaviors of OS cells; miR-494-3p inhibitors could partially abolish the inhibition induced by circ_0081001 knockdown on these malignant phenotypes (Figure 5B–5E). These data implied that the biological function of circ_0081001 was dependent on its regulatory function on miR-494-3p.

### miR-494-3p was capable of directly targeting BACH1

**Figure 6:**
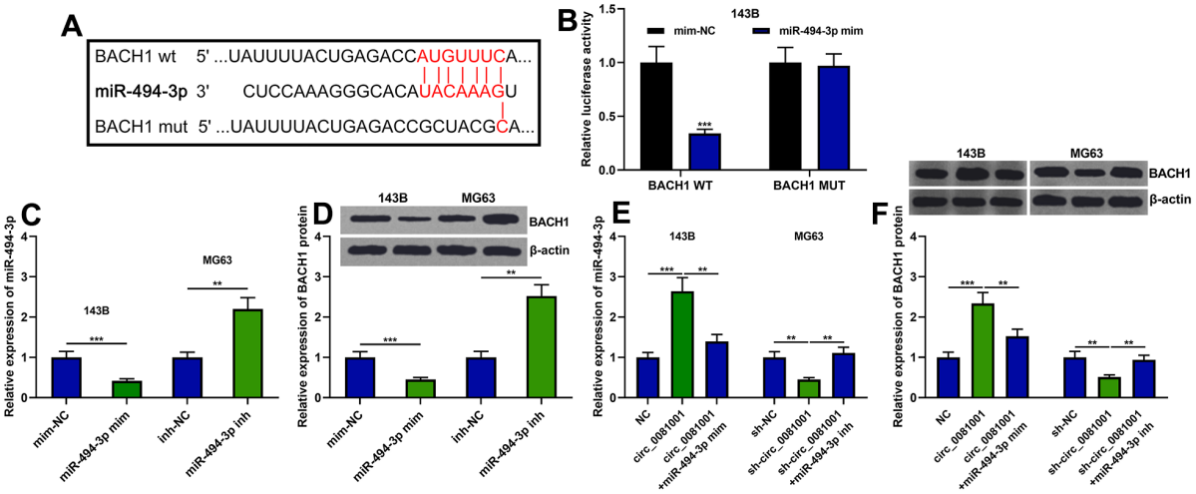
**BACH1 expression was regulated by circ_0081001/miR-494-3p axis.** (**A**) The binding site between miR-494-3p and the 3’UTR of BACH1 mRNA. (**B**) The binding relationship between miR-494-3p and BACH1 mRNA 3’UTR was verified by dual-luciferase reporter gene experiment. (**C**–**F**) The effects of circ_0081001 and miR-494-3p on the expression levels of BACH1 mRNA and protein were examined by qRT-PCR and Western blot after the transfection. ** and *** represent *P*<0.01 and *P*<0.001, respectively.

As mentioned above, a potential regulatory correlation between the miR-494-3p and BACH1 was predicted by bioinformatics analysis (Figures 1C and 6A). Dual-luciferase reporter assay unmasked that miR-494-3p triggered decreased luciferase activity of BACH1-WT but no effect was observed on BACH1-MUT (Figure 6B). Besides, miR-494-3p mimics attenuated BACH1 expression at both mRNA and protein levels while miR-494-3p inhibitors worked oppositely (Figure 6C, 6D). Additionally, circ_0081001 positively regulated BACH1 expression while miR-494-3p counteracted its regulatory function on BACH1 (Figure 6E, 6F). These data authenticated that BACH1 was a target gene of miR-494-3p, and circ_0081001 positively modulated BACH1 expression via suppressing miR-494-3p expression.

## DISCUSSION

CircRNAs feature prominently in the pathogenesis of human diseases, including tumors [4]. Compared with linear RNA, circRNAs, endowed with more stable structures and longer half-life time, possess a promising prospect in the diagnostics and therapeutics of tumors [16]. Several circRNAs are, reportedly, involved in progression of OS. Circ-ITCH expression, for example, is elevated in OS, and circ-ITCH activates PTEN/PI3K/AKT signaling pathway by targeting miR-22, thereupon repressing malignant biological behaviors of OS cells [6]. Circ_0051079 expression is elevated in OS tissues and cell lines, accelerating the biological function of OS cells by modulating miR-26a-5p/TGF-β1 axis [7]. Circ_0081001 is found to be one of the independent diagnostic predictors of OS [10]. In our current research, a significant up-regulation of circ_0081001 expression was observed, which was consistent with the previous reports [10]. Additionally, this work substantiated that circ_0081001 impeded apoptosis and facilitated proliferation, migration, and invasion of OS cells. Our work clarified the biological function of circ_0081001 in OS and defined it as an oncogenic circRNA.

In this work, the downstream mechanism of circ_0081001 and the regulatory relationship between miR-494-3p and circ_0081001 were explored. Reportedly, miR-494-3p partakes in the tumorigenesis and progression of some tumors. MiR-494-3p expression, for instance, is reduced in cervical cancer tissues, and miR-494-3p overexpression suppresses cervical cancer cell proliferation and invasion [17]. Besides, miR-494-3p expression in prostate cancer tissues is observably up-regulated, and a positive correlation between its expression and prostate specific antigen level in the serum of the patients is observed, suggesting that miR-494-3p is a promising biomarker of prostate cancer diagnosis [18]. MiR-494-3p can enhance the inhibitory effect of TNF-related apoptosis-inducing ligand on the process of gastric cancer cells and the promoting effect on apoptosis [19]. Besides, it is reported that miR-494-3p suppresses malignant biological behaviors of OS cells by inhibiting insulin receptor substrate-1 [15]. Additionally, miR-494-3p represses the proliferation and metastasis of U2OS and MG63 cells by targeting CDK6 [20]. In our work, miR-494-3p expression was observed to be observably reduced in OS specimens; the inhibitory effects of miR-494-3p on malignant biological behaviors of OS cells were further validated, and circ_0081001 was proven as a ceRNA for miR-494-3p; moreover, BACH1 was identified as a target gene of miR-494-3p in OS.

BACH1 belongs to the cap'n'collar (CNC) and leucine zipper (b-zip) family [21]. BACH1, widely expressed in various tissues, can regulate fundamental physiological processes by combining with Maf recognition elements [22]. Recent studies report that the aberrant expression of BACH1 affects the progression of multiple cancers. For example, in lung cancer, BACH1 activates the transcription of Hexokinase 2 and GAPDH, enhances the uptake of glucose of cancer cells, improves the glycolysis, and alleviates oxidative stress-induced damage of cancer cells, hence facilitating cancer cell metastasis [21]. In addition, in ovarian cancer, BACH1 boosts the metastasis of cancer cells by enhancing the expressions of Slug, Snail, and HMGA2 [23]. Importantly, a recent study reports that BACH1 is dysregulated in OS tissues, which is mediated by circ_0000337/miR-4458 axis [24]. In our current research, we found that circ_0081001 indirectly augmented BACH1 expression by targeted decreased miR-494-3p expression, thereupon enhancing malignant biological behaviors of OS cells. Our research further clarifies the upstream regulatory mechanism of BACH1 in OS.

## CONCLUSIONS

In summary, our current research unmasks a novel ceRNA network consisting of circ_0081001/miR-494-3p/BACH1 in OS progression, which may offer valuable clues for the diagnosis and treatment of OS.

## MATERIALS AND METHODS

### Tissue samples

Fifty-one OS patients had their cancer tissues extracted. Prior to the surgery, none of these enrolled patients received neoadjuvant treatment such as chemotherapy and radiotherapy. In the control group, the specimens obtained from adjacent tissues (normal cartilage tissues) were examined by postoperative pathological analysis, and no cancerous cells were found.

### Cell culture and cell transfection

Human OS cell lines and osteoblast cell line HFOB1.19, available from the American Type Culture Collection (ATCC, Rockville, MD, USA) and the China Center for Type Culture Collection (CCTCC, Wuhan, China), respectively, were cultured in RPMI-1640 medium (Thermo Fisher Scientific, Waltham, MA, USA) containing 10% fetal bovine serum (FBS, Thermo Fisher Scientific, Waltham, MA, USA), 100 U/mL penicillin, as well as 100 μg/mL streptomycin (Hyclone, Logan, UT, USA) at 37° C in 5% CO_2_. Besides, short hairpin RNA (shRNA) targeting circ_0081001 (sh-circ_0081001), its corresponding negative control (sh-NC), circ_0081001 overexpression plasmid (circ_0081001), and empty vector (negative control, NC) were designed by GeneChem (Shanghai, China). MiRNA-494-3p mimics (miRNA-494-3p mim), miRNA-494-3p inhibitors (miRNA-494-3p inh), and the corresponding negative controls (mim-NC, inh-NC) were available by GenePharma (Shanghai, China). Moreover, Lipofectamine^®^ 2000 reagent was adopted to transfect the cells. The transfection efficiency, after 36 h, was measured employing quantitative real-time polymerase chain reaction (qRT-PCR).

### qRT-PCR

The RNA extraction was performed utilizing TRIzol reagent (Invitrogen, Carlsbad, CA, USA) in compliance with manufacturer’s protocols. The RevertAid™ First Strand DNA Synthesis Kit (Thermo Fisher Scientific, Waltham, MA, USA) was used for reverse transcription. qRT-PCR was conducted with QuantiFast SYBR-Green PCR kit (Roche, Indianapolis, IN, USA) on ABI 7300 Real-time PCR System (Applied Biosystems, Foster City, CA, USA) with cDNA working as the template. The gene relative expressions were determined utilizing 2^-ΔΔCT^ analysis, and the primers were synthesized by BGI (Shenzhen, China) with GAPDH and U6 working as the endogenous controls.

### Cell counting kit-8 (CCK-8) assay

The cells, after being transferred into a 96-well plate (2×10^3^ cells/well) for 1 d, were incubated with 10 μL of CCK-8 kit (Beyotime, Shanghai, China) for 4 h. Then, a microplate reader was adopted to measure the absorbance_450nm_. Similarly, the absorbance of the cells at 48 h and 72 h after the inoculation was determined. Finally, the proliferation curves of the cells in each group were plotted.

### 5-Ethynyl-2'-deoxyuridine (EdU) assay

143B and MG63 cells, after being transferred into a 24-well plate for 1 d, were incubated with 200 μL of 50 for 2 h. The cells rinsed with PBS were fixed with paraformaldehyde for 10 min at ambient temperature and then incubated with 200 μL of glycine (2 mg/mL) for 5 min. After that, the rinsed cells were incubated with 100 μL of PBS containing 0.5% TritonX-100 and then decolorized. Next, the cells were then stained for 30 minutes at ambient temperature in the absence of light with Apollo staining solution, and the nuclei of the cells were then stained for 20 minutes at ambient temperature in the dark with DAPI DNA staining solution. After the rinse with PBS, cell photographing and counting were performed employing a fluorescence microscope.

### Transwell assay

143B or MG63 cells were supplemented to the upper compartment of Transwell system (pore size, 8 μm, 2×104 cells/well; Corning, NY, USA), and meanwhile, 600 μL of medium containing 20% FBS was dripped into the lower one. The cells in the upper compartment were separated after 12 h, and migrated or invaded cells were then fixed with 4% paraformaldehyde, stained with 0.1% crystal violet, and washed with PBS, followed by being dried, photographed, and counted.

### Flow cytometry

Cell apoptosis was quantified employing Annexin V-FTIC/PI Apoptosis Detection Kit (KeyGen, Nanjing, China). OS cells (2×10^5^ cells/well) treated twice with pre-cooled PBS were resuspended in 100 μL of 1×Binding buffer and then incubated with the cell suspension containing 5 μL of AnnexinV-FITC staining solution, together with 5 μL of PI staining solution, at ambient temperature for 15 min in the dark. The apoptosis rate of the cells was quantified employing flow cytometer (BD Biosciences, Franklin Lake, NJ, USA) within 1 h with Flowjo V10 software (BD Biosciences, San Jose, CA, USA) analyzing the data.

### RNA-binding protein immunoprecipitation (RIP) assay

Magna RIP™ RNA-Binding Protein Immunoprecipitation Kit (Millipore, Billerica, MA, USA) was adopted to unmask the correlation between circ_0081001 and miR-494-3p. To put it crudely, the lysis buffer containing RNasin (Takara Biotechnology Ltd., Dalian, China) and protease inhibitor (B14001a, Roche, Basel, Switzerland) was employed to lyse the cells treated with pre-cooled PBS prior to the centrifugation at 12000 g for 30 min. Then, cell lysates were incubated with magnetic beads-coupled with antibody against anti-Argonaute 2 (Ago2) or anti-immunoglobulin G (IgG) (Abcam, Cambridge, UK) at 4° C overnight. Then, proteinase K was incubated with the mixtures, and RNeasy MinElute Cleanup Kit (Qiagen, Shanghai, China) was employed to extract the immunoprecipitated RNA for subsequent qRT-PCR reaction.

### Western blot

OS cells in each group were incubated with pre-cooled RIPA lysis buffer (Beyotime, Shanghai, China) on the ice for 20 min prior to the centrifugation at 13000 r/min at 4° C for 5 min. With supernatants collected as the protein samples, BCA method was employed to quantify the concentration in the samples. After mixing the protein samples with the loading buffer, they were denatured in boiling water for 10 minutes. Next, the polyvinylidene fluoride (PVDF) membrane (Beyotime, Shanghai, China) was employed to transfer the separated protein samples. Then, the membrane incubated with 5% BSA at ambient temperature for 2 h and then with primary antibody anti-BACH1 (ab128486, 1:1000, Abcam, Shanghai, China) at 4° C overnight was rinsed 5 times with TBST and then incubated with secondary antibody [goat anti-mice IgG H&L (HRP) (ab205719, 1:2000, Abcam, Shanghai, China)] for 1 h at ambient temperature. Besides, protein bands were developed employing ECL kit (Amersham Pharmacia Biotech, Little Chalfont, UK) with β-actin utilized as the endogenous control.

### Dual-luciferase reporter gene assay

Wild type (WT) and mutant (MUT) luciferase reporter vectors were constructed and Lipofectamine 2000 (Thermo Fisher Science, Waltham, MA, USA) was adopted to co-transfect miR-494-3p mimics or mim-NC into 143B cells. The luciferase activity was quantified, after 2 d, employing dual-luciferase reporter assay system (Promega, Madison, WI, USA).

### Statistical methods

SPSS16.0 software (SPSS Inc., Chicago, IL, USA) was adopted to analyze the data expressed as mean ± SD (x ± s). The independent sample *t*-test and one-way ANOVA were employed to conduct the comparisons of the data between two groups and amongst multiple groups, respectively. Chi-square test was utilized for the analysis of the correlation between circ_0081001 and patients' pathological characteristics. *P*<0.05 demonstrated statistical significance.

### Ethics statement

Our research was approved by the Ethics Committees of Linyi People's Hospital. The written informed consent was acquired from each participating patient. All protocols were conducted in accordance with the principles of the Declaration of Helsinki.

### Data availability statement

The data used to support the findings of this study are available from the corresponding author upon request.
